# Supplementary material for: A Multidisciplinary Approach in Examining the Susceptibility to Microbial Attack of Polyacrylic and Polyurethane Resins Used in Art Restoration
Source: Int J Mol Sci. 2022 Oct 3;23(19):11725. doi: 10.3390/ijms231911725 (PMC9569816; doi:10.3390/ijms231911725)

Supplementary Figure S1. FT-IR spectra of polyacrylic resins. AC 012, AC 013 and AC P1 controls spectra (a, b, c) and AC 012, AC 013 and AC P1 spectra after 7 days of incubation with fungi (d, e, f).

a)

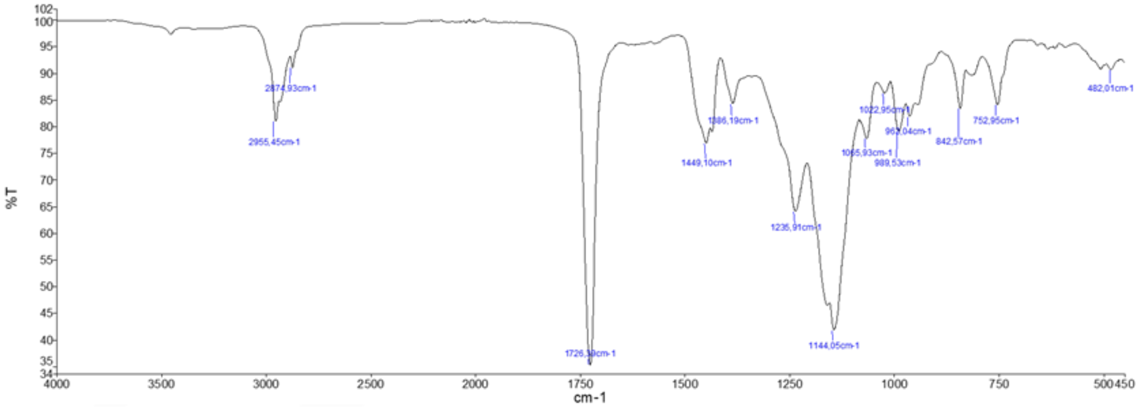

b)

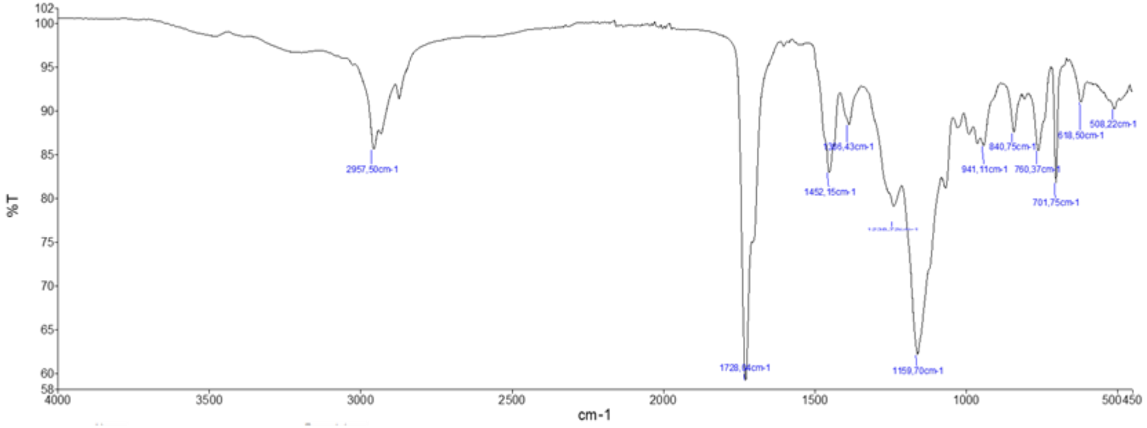

c)

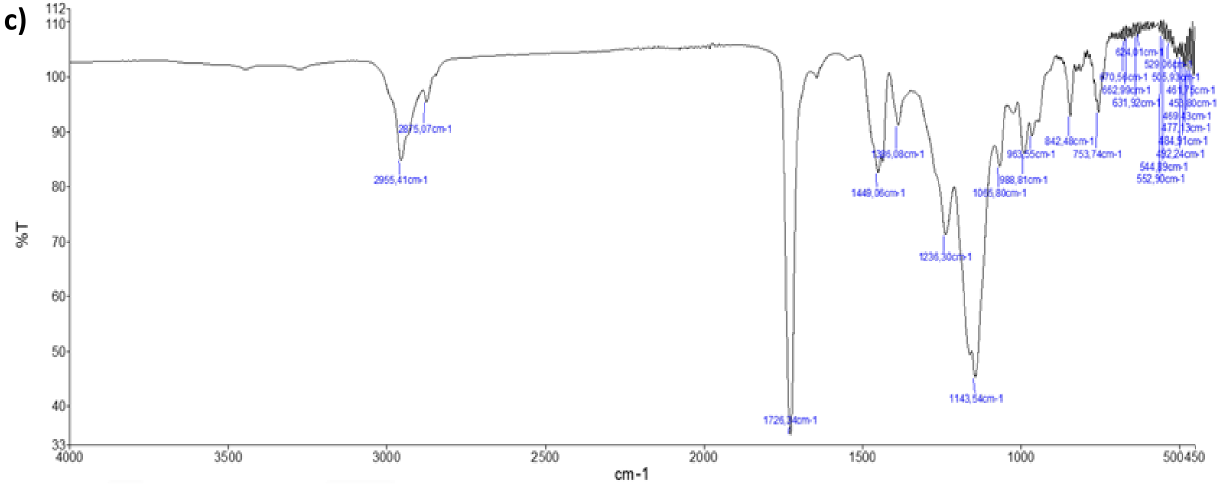

d)

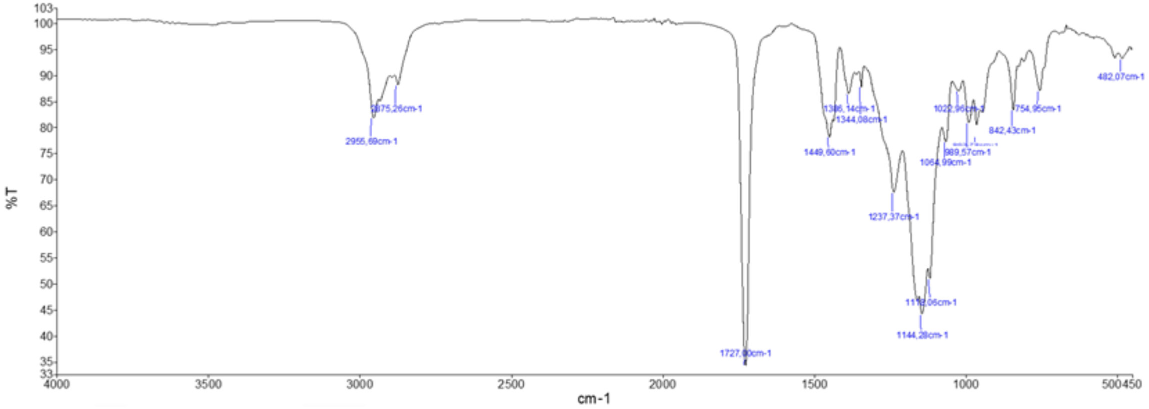

%T
cm-1
2955,69cm-1
2875,26cm-1
1727,90cm-1
1449,60cm-1
1344,08cm-1
1237,37cm-1
1144,28cm-1
1064,99cm-1
1022,96cm-1
989,57cm-1
842,43cm-1
754,96cm-1
482,07cm-1

e)

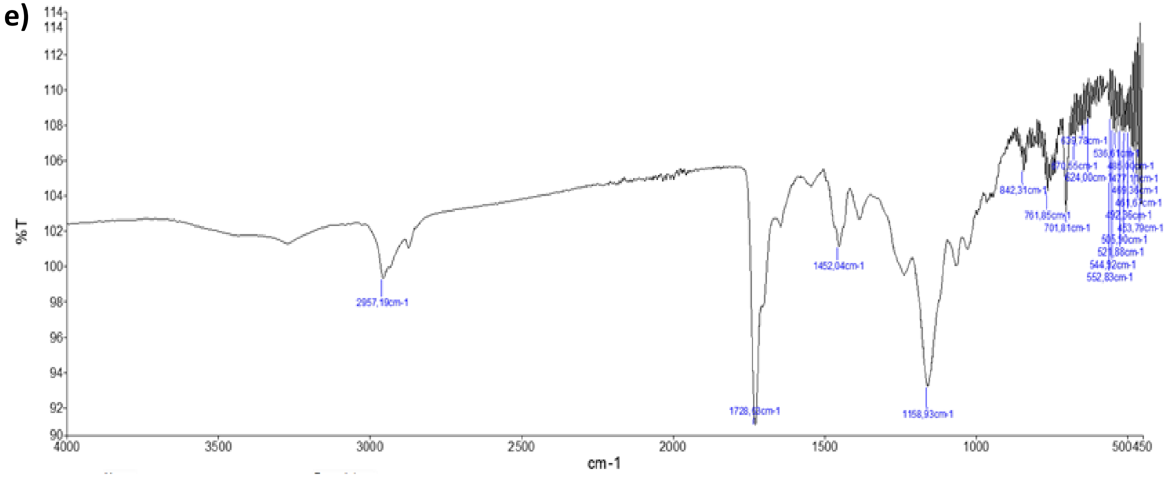

%T
cm-1
2957,19cm-1
1728,63cm-1
1452,04cm-1
1158,93cm-1
842,31cm-1
761,85cm-1
701,81cm-1
624,00cm-1
552,83cm-1

f)

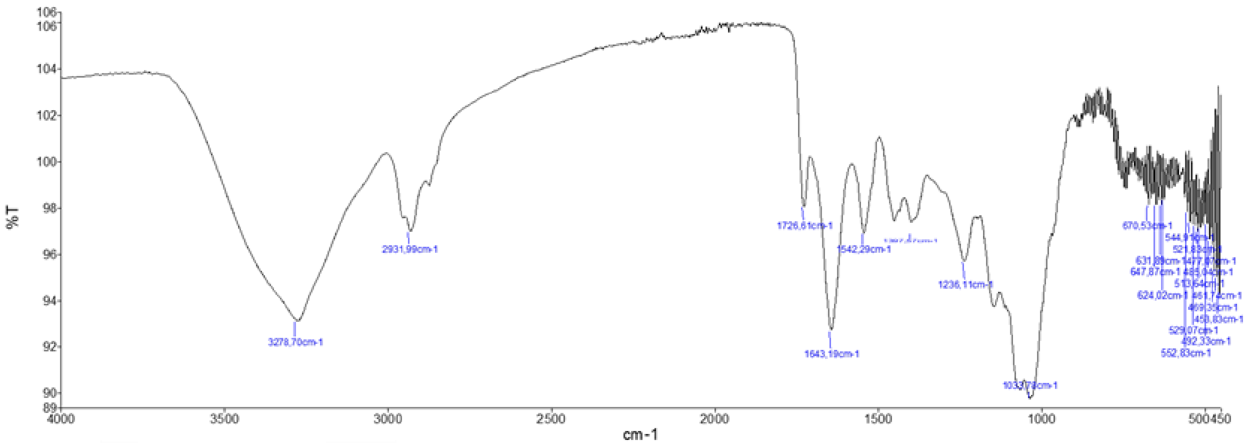

%T
cm-1
3278,70cm-1
2931,99cm-1
1726,61cm-1
1643,19cm-1
1542,29cm-1
1236,11cm-1
670,53cm-1
647,87cm-1
624,02cm-1
552,83cm-1

Supplementary Figure S2. FT-IR spectra of polyacrylic resins AC 012, AC 013, AC P1 and AC 32 after incubation with *P. aeruginosa* ATCC 9027.

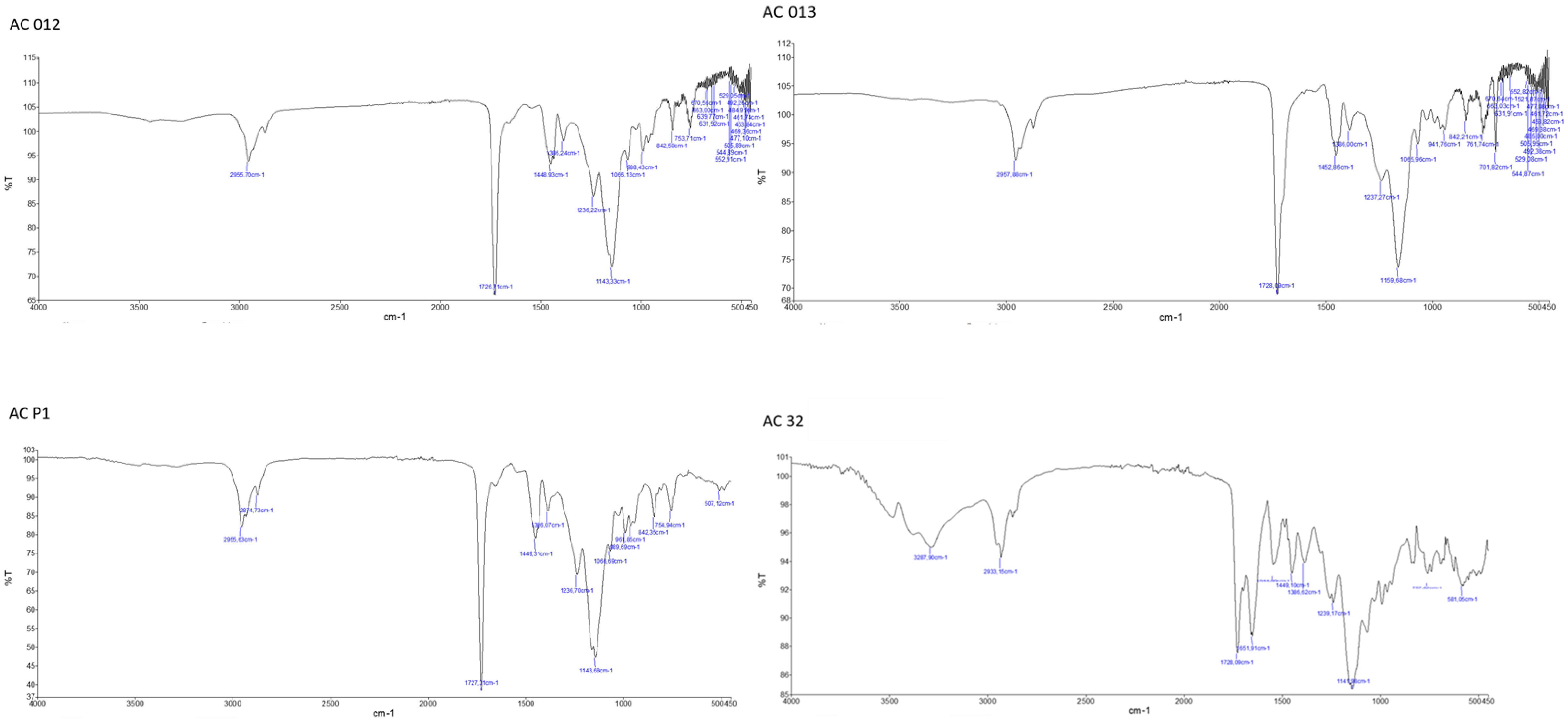

Supplementary Figure S3. FT-IR spectra of polyurethane resins. PU H6 and PU GS controls spectra (a, b) and PU H6 and PU GS spectra after 7 days of incubation with fungi (c, d).

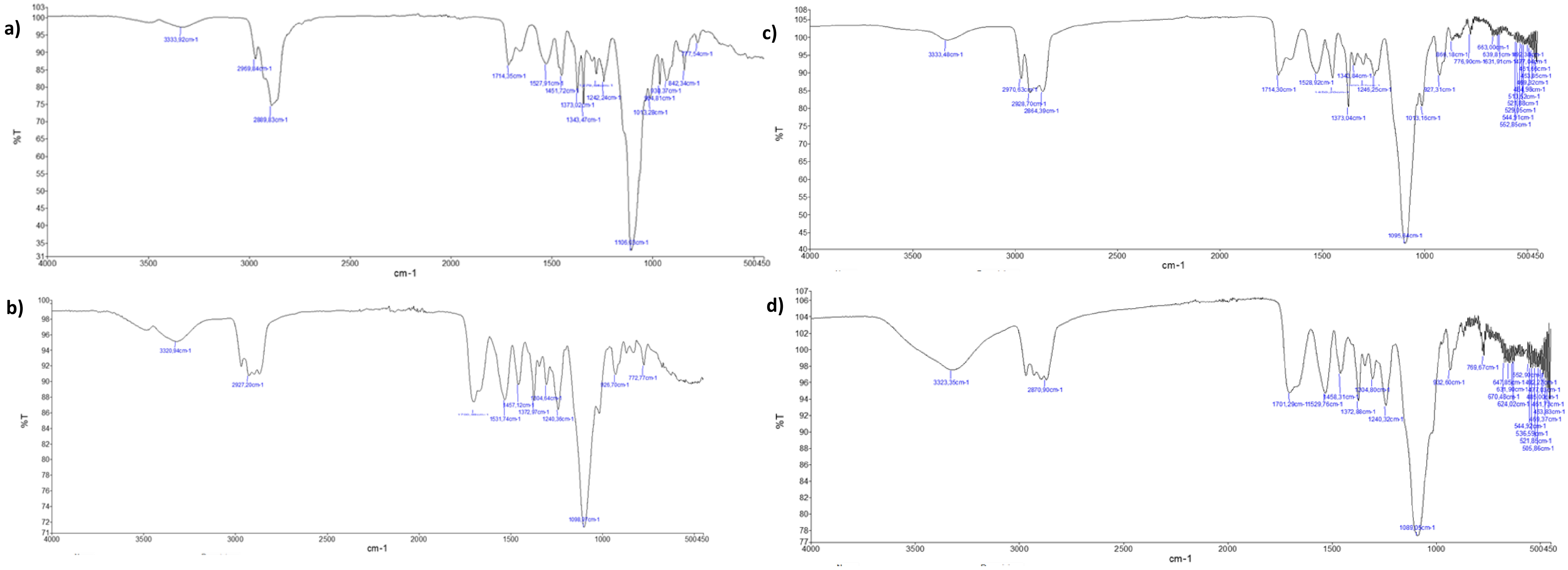

Supplementary Figure S4. FT-IR spectra of polyurethane resin PU GS after incubation with *P. aeruginosa* ATCC 9027.

PU GS

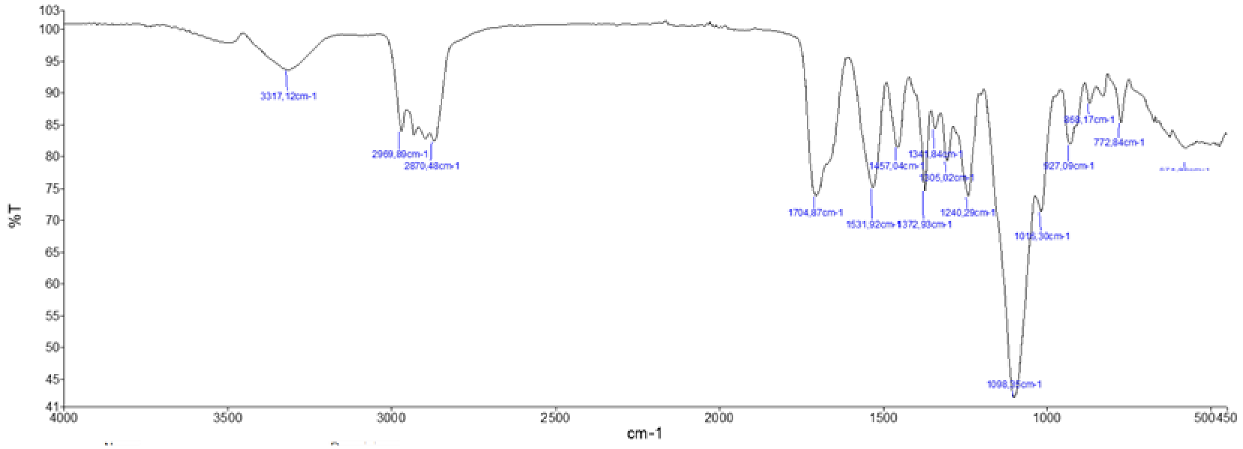

Supplement: Supplementary file 1 [file ijms-23-11725-s001.zip › ijms-1928985-supplementary.pdf]
